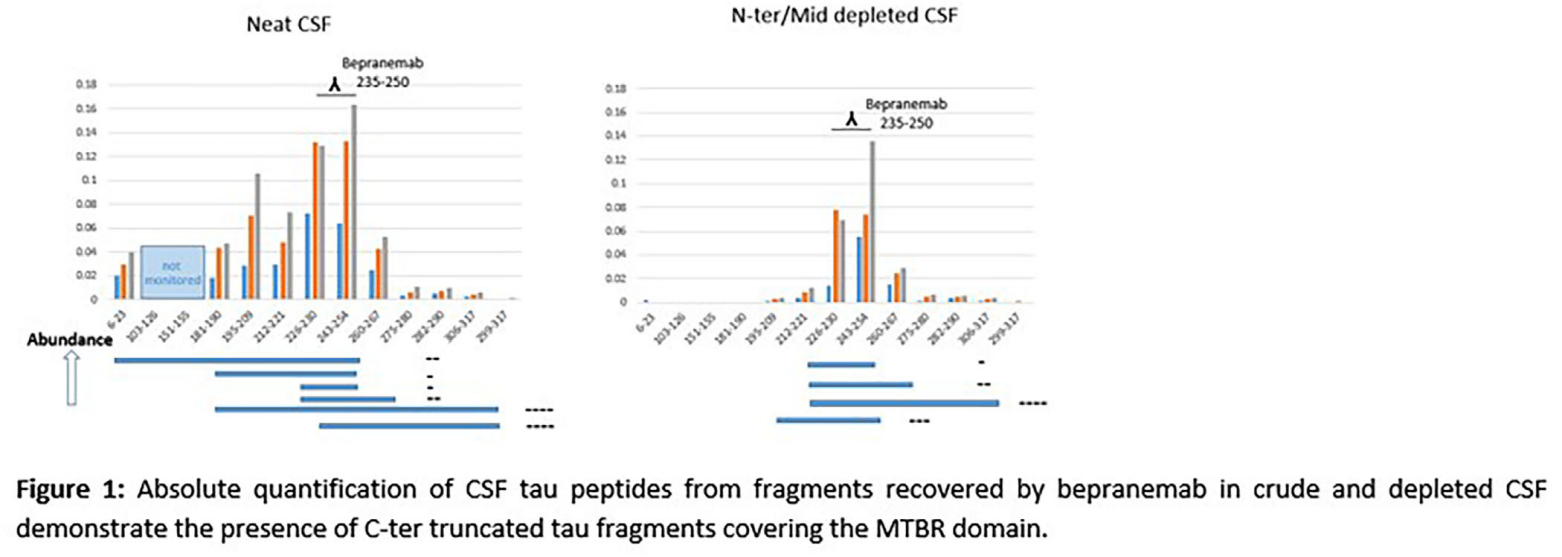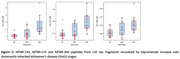# Characterization of cerebrospinal fluid tau MTBR species binding to Bepranemab

**DOI:** 10.1002/alz.089980

**Published:** 2025-01-09

**Authors:** Nicolas R. Barthélemy, Yingxin He, Brendan Androff, Yan Li, Thi Liên‐Anh Nguyên, Patrick Downey, Eric McDade, Randall J. Bateman

**Affiliations:** ^1^ Washington University School of Medicine, St. Louis, MO USA; ^2^ The Tracy Family SILQ Center, St. Louis, MO USA; ^3^ UCB Pharma, Braine‐l'Alleud Belgium; ^4^ The Tracy Family SILQ Center, Washington University School of Medicine, St. Louis, MO USA

## Abstract

**Background:**

Alzheimer’s disease neuropathology involves the deposition in brain of aggregates enriched with microtubule‐binding‐region (MTBR) of tau adopting an abnormal conformation between residues 306‐378 in the core of aggregates. Anti‐tau drugs targeting around this domain have the potential to interfere with the cell‐to‐cell propagation of pathological tau. Bepranemab is a humanized monoclonal Ig4 antibody binding to tau residues 235‐250. Previous studies showed this antibody binds to tau monomers; inhibits tau seeding in cellular assay and prevents tau pathology spread in a mouse model. The target engagement of bepranemab in human biofluids is unknown. This study aims to characterize cerebrospinal fluid (CSF) tau species binding to this antibody.

**Methods:**

We used mass spectrometry (MS) to characterize CSF tau species precipitating with bepranemab cross‐linked to sepharose beads. Precipitated CSF tau species were digested with trypsin and tau peptides were quantified by signal comparison to peptides from tau labeled standard. We characterized and compared the amount of bepranemab‐reactive tau species recovered from neat CSF and from CSF initially depleted from N‐terminal and Mid‐domain truncated tau species. Then, we analyzed Bepranemab precipitates in CSF samples from 14 asymptomatic dominantly inherited Alzheimer’s disease (DIAD) mutation carriers (aMC), 16 symptomatic (sMC) and 15 non‐carriers (NC).

**Results:**

Bepranemab bound to a mixture of CSF N‐terminal and Mid‐domain tau‐truncated fragments including mainly 226‐254 tau residues on their C‐terminal domain (Figure 1). Bepranemab‐reactive species not containing the N‐ter and the Mid‐domain of tau were mostly comprised of fragments including tau residues 226 to 267. Minor bepranemab‐reactive tau species contained tau residues 275 to 290 on their C‐terminal. Tau peptides after residues 317 were not detected above the MS limit of detection. In DIAD, the pool of bepranemab‐immunoreactive CSF tau fragments significantly increased in asymptomatic and symptomatic mutations carriers respectively (Figure 2).

**Conclusions:**

The pool of CSF tau species included a subset of tau MTBR‐containing fragments recovered by bepranemab. A minor fraction of these fragments had domains overlapping with the upstream domain constituting the AD tau aggregate core. These fragments increased over DIAD stages. Our study supports bepranemab engagement against a specific pool of tau species in CSF. This work was sponsored by UCB.